# Identification of miRNAs Present in Cell- and Plasma-Derived Extracellular Vesicles—Possible Biomarkers of Colorectal Cancer

**DOI:** 10.3390/cancers16132464

**Published:** 2024-07-05

**Authors:** Marzena Lenart, Izabela Siemińska, Rafał Szatanek, Anna Mordel, Antoni Szczepanik, Mateusz Rubinkiewicz, Maciej Siedlar, Monika Baj-Krzyworzeka

**Affiliations:** 1 Department of Clinical Immunology, Medical College, Jagiellonian University, 30-663 Krakow, Poland; m.lenart@uj.edu.pl (M.L.); izabela.sieminska@urk.edu.pl (I.S.); rafal.szatanek@uj.edu.pl (R.S.); misiedla@cyf-kr.edu.pl (M.S.); 2 Institute of Veterinary Sciences, University Center of Veterinary Medicine JU-AU, University of Agriculture in Krakow, 30-059 Krakow, Poland; 3 Department of Clinical Immunology, University Children’s Hospital of Cracow, 30-663 Krakow, Poland; amordel@usdk.pl; 4 Third Department of Surgery, Faculty of Medicine, Jagiellonian University Medical College, 31-202 Krakow, Poland; antoni.szczepanik@uj.edu.pl; 5 Second Department of Surgery, Jagiellonian University Medical College, 30-688 Krakow, Poland; mateusz.rubinkiewicz@uj.edu.pl

**Keywords:** colorectal cancer, tumor-derived extracellular vesicles, miRNAs, miRNA expression profile, colorectal cancer cell lines

## Abstract

**Simple Summary:**

Globally, colorectal cancer is the third and second most common cancer in men and women, respectively. Due to its late diagnosis, morbidity and mortality are on the rise, which legitimizes the search for new markers for early and precise detection of cancer development. The knowledge about the short, non-coding RNA molecule (microRNA) expression profile in tumor cancer cell lines and extracellular vesicles derived from them may help to establish microRNA as useful markers for monitoring tumor signatures in body fluids (i.e., blood). Here, we present a comprehensive analysis of microRNA profiles of four colorectal cancer cell lines, one normal colon epithelium cell line and extracellular vesicles derived from them. Data are supplemented with preliminary results acquired from a group of colorectal cancer patients.

**Abstract:**

Globally, an increasing prevalence of colorectal cancer (CRC) prompts a need for the development of new methods for early tumor detection. MicroRNAs (also referred to as miRNAs) are short non-coding RNA molecules that play a pivotal role in the regulation of gene expression. MiRNAs are effectively transferred to extracellular vesicle (EVs) membrane sacs commonly released by cells. Our study aimed to examine the expression of miRNAs in four CRC cell lines and EVs derived from them (tumor EVs) in comparison to the normal colon epithelium cell line and its EVs. EVs were isolated by ultracentrifugation from the culture supernatant of SW480, SW620, SW1116, HCT116 and normal CCD841CoN cell lines and characterized according to the MISEV2023 guidelines. MiRNAs were analyzed by small RNA sequencing and validated by quantitative PCR. The performed analysis revealed 22 common miRNAs highly expressed in CRC cell lines and effectively transferred to tumor EVs, including miR-9-5p, miR-182-5p, miR-196b-5p, miR-200b-5p, miR-200c-3p, miR-425-5p and miR-429, which are associated with development, proliferation, invasion and migration of colorectal cancer cells, as well as in vesicle maturation and transport-associated pathways. In parallel, normal cells expressed miRNAs, such as miR-369 and miR-143, which play a role in proinflammatory response and tumor suppression. The analysis of selected miRNAs in plasma-derived EVs and tumor samples from CRC patients showed the similarity of miRNA expression profile between the patients’ samples and CRC cell lines. Moreover, miR-182-5p, miR-196-5p, miR-425-5p and miR-429 were detected in several EV samples isolated from patients’ plasma. Our results suggest that miR-182-5p, miR-196b-5p and miR-429 are differentially expressed between EVs from CRC patients and healthy donors, which might have clinical implications.

## 1. Introduction

Colorectal cancer (CRC) is the third most diagnosed cancer in the world [1]. In 2020, 1.93 million new cases of CRC were diagnosed, of which 0.94 million patients died, while in 2040, the global number of new cases of this type of cancer is predicted to reach 3.2 million [2]. CRC is associated with high mortality; therefore, the best method to reduce the frequency and negative effects of this disease is its early diagnosis. For example, commonly available colonoscopy reduces CRC incidence by about 40% and mortality by about 60% [3]. Considering these statistics, there is still a need for the development of new screening methods that are fast, reliable and non-invasive. One of the options for such approaches involves the analysis of circulating extracellular vesicles (EVs) [4].

EVs are cell-derived, membrane-bound vesicles that are generated by different types of normal and malignant cells. EVs contain biomolecules originating from their parent cells that can be internalized by other cells, making EVs important conveyors in intercellular communication [5]. EVs transfer bioactive cargo, including cell surface or cytoplasmic proteins, lipid rafts, mRNA or non-coding RNAs, including microRNA (miRNA, also called miR) [6,7,8]. Thus, EVs, depending on the transported cargo, might even exert negative effects by transmitting signals leading to the suppression of antitumor immune responses. It has been shown that EVs play a role in the establishment and maintenance of tumor microenvironment by sustaining cell proliferation, evading growth suppression, resisting cell death, acquiring genomic instability and reprogramming stromal cell lineages, which, altogether, contributes to its functional remodeling [9,10]. 

miRNAs are short non-coding RNA molecules that play a regulatory role in gene expression by repressing translation or cleaving RNA transcript [11]. MiRNAs are being considered as possible biomarkers in clinical diagnosis of cancer, disease progression monitoring or even in treatment development [12]. The current field of interest focuses on tumor-derived EVs transporting tumor-associated miRNAs, with CRC being at the forefront of these studies. For example, miR-21, the most widely studied miR in EVs, has been suggested as a promising prognostic and recurrence biomarker in CRC [13]. The treatment of CRC cells with miR-21 led to an increased expression of genes that were involved in cell proliferation, invasion and extracellular matrix formation [14]. miRNAs, both circulating and transported by EVs in body fluids of CRC patients, may have a diagnostic value as a predictor of CRC development. Chen et al. showed 14 miRNAs uniquely detected in the serum of CRC patients, which included miR-485-5p, miR-361-3p, miR-326 and miR-487b [15]. Others indicated that plasma-derived miR-29a and miR-92a showed a strong potential as novel non-invasive biomarkers for early detection of CRC [16]. Several studies identified miRNAs candidates for biomarkers of early CRC diagnosis. As such, let-7a, miR-1229, miR-1246, miR-150, miR-21, miR-223 and miR-23a were significantly higher in CRC patients, including early stages of the disease, than in healthy controls [17]. Wang et al. showed that miR-125a-3p and miR-320c were significantly upregulated in plasma exosomes of patients with early-stage CRC [18]. Finally, RNA sequencing analysis on plasma EVs derived from 50 healthy individuals and 142 cancer patients revealed five potential EVs-transported miRNAs as potential biomarkers, that is miR-1343-3p, miR-125a-5p, miR-708-5p, miR-381-3p and miR-543 [19]. These examples show that although many EV-bound miRNAs might be associated with CRC development, a definitive bio-marker of the disease is still unknown. Recently, Chen et al., in an elegant study concerning CRC cell lines-based microRNA signatures, indicated that EV-transported miR-7641 may represent a reliable candidate for a specific diagnostic and prognostic biomarker of CRC [20]. Thus, considering the ongoing work concerning CRC biomarker discovery, including the microRNAs potential in this matter, not only justifies further research in this field but also makes it that more essential.

Here, we performed differential analysis of miRNA profiles of tumor cells of four CRC cell lines and the EVs derived from them. The analysis revealed a set of 22 miRNAs that were commonly expressed and efficiently loaded into their EVs, yet not expressed in cells of the control normal epithelium cell line. Finally, we undertook the challenge to validate the expression of selected miRNA in EVs and solid tumor samples obtained from CRC patients. We observed similarities in the miRNA expression profile between CRC cell lines and patients’ tumor samples and detected CRC-related miRNAs in EVs isolated from patients’ plasma. 

## 2. Materials and Methods

### 2.1. Cell Culture and EVs Isolation

Human CRC cell lines were purchased from ATCC (Manassas, VA, USA) and cultured according to the following ATCC recommendations: SW480 (CCL-228), SW620 (CCL-227), SW-1116 in Leibovitz’s medium L-15; HCT116 in McCoy’s 5A medium and normal epithelium CCD841CoN cell line in αMEM; all supplemented with L-glutamine (PAN Biotech, Aidenbach, Germany). Cells were cultured in 175-cm^2^ flasks in an appropriate medium supplemented with 10% (*v*/*v*) EV-depleted, low-endotoxin fetal bovine serum (Biowest, Nuaille, France) and 1% (*v*/*v*) Penicillin Streptomycin (Pen/Strep, PAN Biotech) at 37 °C and 5% CO_2_ (except CO_2_-free L-15 cultures). Cells were regularly tested for Mycoplasma sp. contamination (Abcam, Cambridge, UK). Culture supernatants were collected for EV isolation from cell cultures with a confluency of above 90%. Briefly, to remove cells and cellular debris, supernatants were centrifuged at 500× *g* for 10 min, followed by 3200× *g* for 15 min. Next, the supernatants were ultracentrifuged at 100,000× *g* for 2 h in a Sorvall™ WX+ Ultracentrifuge equipped with the T-1270 rotor (Thermo Fisher Scientific Inc., Waltham, MA, USA). EV pellets were washed twice in filtered PBS and used for further analysis.

### 2.2. Patients and EVs Isolation

A group of 9 patients (4 female and 5 males, with an average age of 64.8 years old) across various stages of CRC (characterized in Supplementary Table S2) was enlisted from Departments of General Surgery at Jagiellonian University Medical College in Krakow. Approximately 4.8 mL of whole blood samples were collected from both patients and healthy donors. Blood from healthy donors was purchased from the Regional Center of Blood Donation and Blood Therapy (Krakow, Poland; agreement no. DZM/SAN/CM/U-678/2015). Subsequently, from these blood samples, approximately 1 mL of plasma was obtained. Additionally, to the collected whole blood samples, solid tumor samples were obtained from the same patients. Ethical considerations were paramount throughout the study, with all procedures receiving approval from the Jagiellonian University Bioethics Committee (approval numbers: 1072.6120.96.2022, 1072.6120.323.2021 and 1072.6120.324.2021). Prior to their involvement, all participants provided written informed consent, affirming their willingness to contribute to the study. Concurrently, a control group comprising 9 healthy adult blood donors was recruited.

EVs were isolated from 1 mL of each patient/donor plasma sample by the ultracentrifugation method described above. Briefly, to remove cells and cellular debris, the plasma samples were centrifuged at 500× *g* for 10 min followed by 10,000× *g* for 30 min. Next, the samples were ultracentrifuged at 100,000× *g* for 2 h in a Sorvall™ WX+ Ultracentrifuge with T-1270 rotor (Thermo Fisher Scientific Inc.). EV pellets were washed twice in filtered PBS and used for further analysis.

Solid tumor samples from patients were collected, ground with a scalpel and passed through a 40 µm strainer. After rinsing in PBS, the cells were frozen in RL lysis buffer (EurX, Gdansk, Poland) and used later for RNA isolation. 

### 2.3. Characterization of EVs

EVs isolated from culture supernatants (HCT116, SW1116, SW480, SW620 and CCD841CoN) and from plasma of CRC patients and healthy donors were characterized by size, concentration, expression of common markers and membrane structure. The size and concentration of EVs were determined by Nanoparticle Tracking Analysis (NTA) using the PMX-230 (Twin) ZetaView (Particle Metrix GmbH, Inning am Ammersee, Germany). Prior to its use, the ZetaView analyzer was calibrated using 100 nm polystyrene beads (Applied Microspheres B.V., Leusden, The Netherlands). For sample measurements, EVs were diluted 1000 times in 1 mL filtered (0.2 μm), sterile 1x PBS. Next, the prepared EV samples were aspirated into an insulin syringe and loaded into the sample measuring chamber of the ZetaView analyzer, where the measurements were performed. To obtain the size, size range and concentration of the analyzed EVs, the collected data were processed using the ZetaView analysis software, version 8.05.16SP3 (Particle Metrix GmbH).

EVs membrane structure was confirmed by MEMGlow staining. Briefly, MEMGlow solution (20 μM; Cytoskeleton, Inc., Denver, CO, USA) was diluted to 0.1 μM concentration and incubated with EVs for 30 min at RT in the dark. Following the incubation, the stained EVs, without washing, were analyzed by flow cytometry using the CytoFLEX™ cytometer (Beckman Coulter, Indianapolis, IN, USA).

### 2.4. Western Blotting (WB)

Proteins from EVs were extracted with the RIPA lysis buffer (Thermo Fisher Scientific, Waltham, MA, USA) supplemented with protease and phosphatase inhibitor cocktail (Thermo Fisher Scientific), and their concentration was determined using the Bradford method according to the standard protocol and measured with the DS-11 Spectrophotometer (DeNovix, Wilmington, DE, USA). A 20 μg/sample of EV proteins was mixed with LDS Sample Buffer and Sample Reducing Agent (Thermo Fisher Scientific) and incubated at 75 °C for 10 min, after which, the samples were placed on ice. Next, the EV samples were loaded into the wells of the 4% polyacrylamide stacking gel and electrophoresed at 180 V for 45 min in the 14% polyacrylamide resolving gel. Then, EVs proteins were transferred from the gel onto a polyvinylidene fluoride membrane (Merck Millipore, Burlington, MA, USA) with semi-dry transfer at 25 V for 1 h, blocked at RT for 1 h with 2% bovine serum albumin in Tris-buffered saline with 0.1% Tween (1× TBST + 2% BSA) and incubated overnight at 4 °C with either rabbit anti-CD9 (clone D8O1A, Cell Signaling Technology, Danvers, MA, USA), rabbit anti-CD63 (polyclonal IgG, Proteintech, Group, Chicago, IL, USA) or rabbit anti-GM130 (clone D6B1, Cell Signaling Technology). Following the overnight incubation, the membranes were incubated for 1 h at RT with an anti-rabbit IgG (Cell Signaling Technology) secondary antibody conjugated with horseradish peroxidase. Next, the protein bands were visualized with Clarity Max™ Western ECL Substrate (Bio-Rad Laboratories, Hercules, CA, USA) using the G: BOX Chemi-XR5 gel imaging system (Syngene, Cambridge, UK) equipped with GeneSys v.1.2.5.0 software and their mass was assessed using the color-coded pre-stained protein marker (Cell Signaling Technology).

### 2.5. Total RNA Isolation

Total RNA was isolated from cell lines, solid tumor samples and EVs using commercially available kits mirVana™ miRNA Isolation Kit and Total Exosome RNA & Protein Isolation Kit, respectively (both from Ambion, Life Technologies, Austin, TX, USA). Total RNA obtained from EVs before further processing required concentration, which was carried out using the GeneJET RNA Cleanup and Concentration Micro Kit (Thermo Fisher Scientific) according to the manufacturer’s protocol. The final volume of concentrated total RNA was 10 µL.

### 2.6. MicroRNA Next-Generation-Sequencing

The miRNA expression profile was analyzed using deep sequencing performed by TAmiRNA GmbH (Vienna, Austria). Briefly, a total of 85 ng RNA from cell lines was used as an input, while in the case of EV samples, a defined input volume of 8.5 µL suspension was used. To each RNA sample, 1 µL of mind spike-in standards (TAmiRNA, Vienna, Austria) was added. Small RNA library preparation was performed using the RealSeq Biofluids library preparation kit (RealSeq Biosciences, Santa Crus, CA, USA), and then, libraries were amplified using barcoded Illumina reverse primers in combination with the Illumina forward primer (21 cycles for cellular RNA, 24 cycles for EV RNA). Library quality control was performed using the DNA1000 Chip (Agilent, Santa Clara, CA, USA). RNA sequencing was performed on the Illumina NextSeq 550 with 75 bp single-end reads [21]. NGS data were processed using the miND pipeline [21]. The quality of the data was evaluated with fast QC v0.11.8 [22] and multiQC v1.7 [23], filtered using cutadapt v2.3 [24] for a minimum length of 17 nt. Mapping steps were performed with bowtie v1.2.2 [25] and miRDeep2 v2.0.1.2 [26] against the genomic reference GRCh38.p12 provided by Ensembl [27] and, subsequently, using miRBase v22.1 [28].

To identify miRNAs enriched in tumor cell lines and their EVs, we first filtered miRNAs that expressed no more than 30 transcripts per million reads. Then, differentially expressed miRNA content in analyzed cell lines and tumor EVs were analyzed and presented using Venn diagrams (online tool provided by Bioinformatics & Evolutionary Genomics, University of Gent, Gent, Belgium, https://bioinformatics.psb.ugent.be/webtools/Venn, accessed on 25 June 2024) or by hierarchical clustering analysis (Morpheus, Broad Institute, USA, https://software.broadinstitute.org/morpheus, accessed on 25 June 2024). GO (Gene Ontology) enrichment and KEGG (Kyoto Encyclopedia of Genes and Genomes) pathway analysis of targeted genes were performed using DIANA-miRPath v4.0 with target selection from TarBase v.8.0 and second annotation TargetScan v.8.0 [29]. Differentially expressed genes were selected with *p*-value < 0.05. To visualize the network of selected miRs and their targets, miRNet 2.0 was used [30] based on the miRTarBase v.8.0.

### 2.7. qRT-PCR

Quantitative real-time PCR (qRT-PCR) was used to analyze the expression levels of selected miRNAs and to validate NGS results. Briefly, total RNA from cells (100 ng) and EVs (10 ng) was obtained as described above, transcribed into cDNAs using reverse transcription (RT) primers using a TaqMan™ MicroRNA Reverse Transcription Kit (Thermo Fisher Scientific, Inc.) with the thermal profile: 30 min at 16 °C, 30 min at 42 °C, 5 min at 85 °C. qPCR reactions were performed in triplicate using specific primers and probes (hsa-miR-9-5p ID 000583, hsa-miR-19a-3p ID 000395, hsa-miR-143-3p ID 002249, hsa-miR-146a-5p ID 000468, hsa-miR-181a-5p, hsa-miR-182-5p ID 002334, miR-196b-5p ID 002215, hsa-miR-200b-5p ID 002274, hsa-miR-200c-3p ID 002300, hsa-miR-369-3p ID 000557, hsa-miR-425-5p ID 001516 and hsa-miR-429 ID 001024, all from Thermo Fisher Scientific), TaqMan Universal PCR MasterMix (Thermo Fisher Scientific) and QuantStudio 7 System (Applied Biosystems, Waltham, MA, USA). The PCR reaction thermal profile was as follows: 2 min at 50 °C, 10 min at 95 °C and 15 s at 95 °C and 1 min at 60 °C for 40 cycles. The fluorescent signals generated during the informative log-linear phase were used to calculate the relative amount of miRs, while U6 (ID 001973) was used as a control for each PCR run. The expression of each miRNA was calculated using the 2^−ΔΔCt^ method.

### 2.8. Statistical Analysis

Statistical analysis was performed using GraphPad Prism version 6 (GraphPad Software Inc., San Diego, CA, USA). Data were analyzed using the Mann–Whitney test or Kruskal–Wallis test with Dunn’s post hoc test. The median with interquartile range (IQR) was shown. The *p*-values <0.05 were considered significant.

## 3. Results

### 3.1. Characterization of EVs

**Figure 1:**
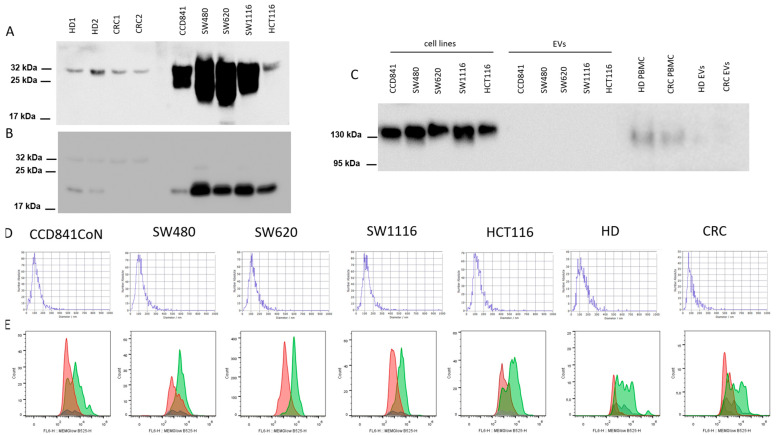
EV characterization. WB images of CD9 (**A**) and CD63 (**B**) protein expression in EV lysates obtained from healthy donors (HD), colorectal cancer (CRC) patients and cell lines; lanes from left to right: 1-2-HD, 3-4-CRC patients, 5-protein ladder, 6-CCD841CoN, 7-SW480, 8-SW620, 9-SW1116 and 10-HCT116. Molecular weights for CD9 are 22, 24 and 35 kDa, and for CD63-28–35 kDa. WB image of GM130 (**C**) protein expression in cell line lysates and their EVs, HD and CRC peripheral blood mononuclear cells (PBMC) and EVs lysates; lanes from left to right: 1-CCD841CoN, 2-SW480, 3-SW620, 4-SW1116, 5-HCT116, 6-CCD841CoN EVs, 7-SW480 EVs, 8-SW620 EVs, 9-SW1116 EVs, 10-HCT116 EVs, 11-protein ladder, 12-HD PBMC, 13-CRC PBMC, 14-HD EVs and 15-CRC EVs. The molecular weight for GM130 is 140 kDa. The uncropped blots and molecular weight markers are shown in Supplementary Figure S1. The size profile of EVs (results from one representative measurement for each case are shown) measured using NTA (**D**); MEMGlow staining of EVs plasma membrane (results from one representative measurement for each case are shown) (**E**). For (**D**,**E**), graphs from left to right: cell lines: CCD841CoN, SW480, SW620, SW1116 and HCT116; HD, CRC patients. Color legend: green—stained EVs, red—unstained EVs, gray—PBS with and without MEMGlow.

NTA analysis of EVs revealed that the average sizes, modal values, size ranges and concentration of all the different types of evaluated EVs were comparable with each other. For HCT116 EVs, the average size was 137.3 nm, and the modal value was 105.9 nm; for SW1116 Evs, it was 127.2 and 112.3 nm; for SW480 Evs, it was 130.1 and 104.5 nm; and for SW620 Evs, it was 133.8 and 104.6 nm. For EVs derived from CCD841CoN cells, it was 125 and 104.4 nm, respectively. The size range for all the evaluated EVs was in the range of 30–420 nm. The concentrations obtained for the different types of EVs were as follows: for HCT116 tumor EVs—1.9 × 10^11^/mL, for SW1116—3.2 × 10^11^/mL, for SW480—2.4 × 10^11^/mL, for SW620—2.1 × 10^11^/mL and for CCD841CoN—2.7 × 10^11^/mL. The average size of EVs (n = 9) isolated from healthy donors’ plasma was 116.5 nm, and from the plasma of CRC patients, it was 116.3 nm. The concentration of EVs was 4.85 × 10^13^/mL and 3.5 × 10^13^/mL for donors and CRC patients, respectively. The membrane structure of EVs was confirmed using MEMGlow staining and WB analysis revealed the presence of both CD9 and CD63 proteins in the different types of evaluated EVs with the absence of the GM130 negative marker. Representative results for WB, NTA and MEMGlow are presented in Figure 1.

### 3.2. miRNA Enrichment in Tumor Cell Lines and EVs Derived from Them

**Figure 2:**
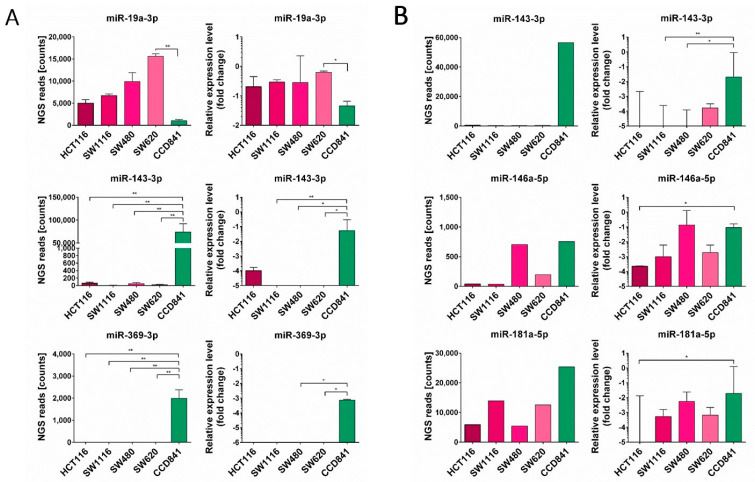
Validation of NGS results (left panel) of selected miRNA expression using qPCR (right panel) in the cell lines (**A**) or EVs (**B**). MiRNA expression of miR-19a-3p, miR-143-3p and miR-369-3p in cell lines (**A**) or the expression of miR-143-3p, miR-146a-5p and miR-181a-5p in EVs (**B**) was analyzed using RT-qPCR and normalized to U6 expression using the 2^−ΔΔCT^ method. Data were analyzed using the Kruskal–Wallis test with Dunn’s post hoc test, and medians ± IQR are shown. * *p* < 0.05, ** *p* < 0.01.

To identify miRNAs enriched in tumor cell lines and their EVs, we performed a pilot miRNA NGS analysis of CRC cell lines (SW480, SW620, SW1116 and HCT116) and their EV samples, and compared the results with those of normal CCD841CoN cell line and EVs derived from it. NGS analysis was performed in duplicate in the case of cell lines and once for EVs samples. Based on the obtained NGS reads (Table S1), we then excluded miRNAs that had less than 30 reads in all analyzed samples and obtained 164 miRNAs for further analysis. NGS data validation was performed using qPCR analysis of selected miRNA expression in both cell lines and their EVs (Figure 2).

**Figure 3:**
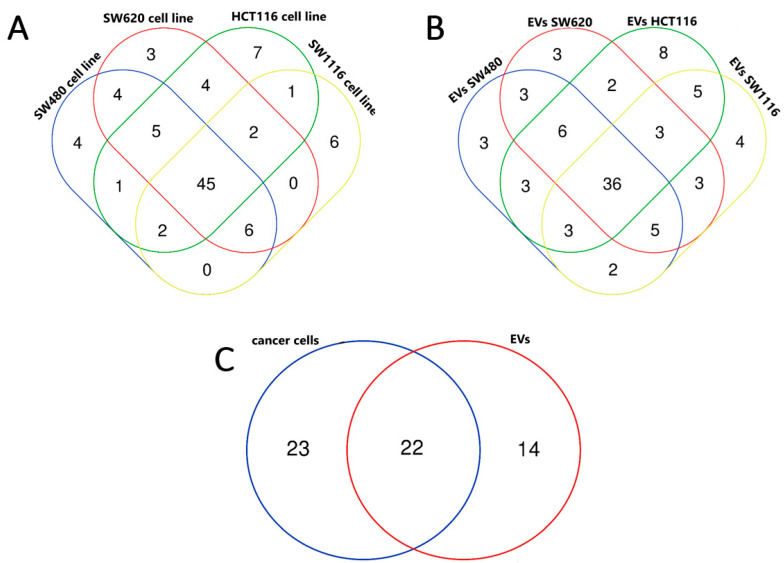
The differential expression of miRNAs using Venn diagrams. Comparison of miRNAs that were at least two times more expressed in tumor cell lines, compared to the normal CCD841CoN cell line (**A**) and in tumor EVs, compared to EVs isolated from control CCD841CoN cell line (**B**). Common miRNAs, showing 2FC difference for both tumor cell lines and tumor EVs, compared to the normal cell line or its EVs, respectively (**C**).

**Figure 4:**
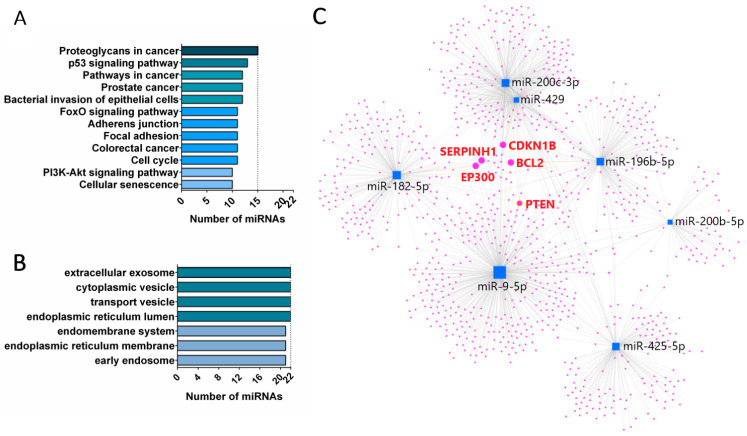
KEGG and GO analysis of selected top 22 miRNAs and visualization of targeted genes. KEGG pathway analysis results of the top 12 pathways associated with at least 10 analyzed miRNAs (**A**). GO analysis of cellular components showing 22 analyzed miRNAs significantly associated with EVs development and processing (**B**). Visualization of genes targeted by selected seven miRNAs: miR-9-5p, miR-196b-5p, miR-182-5p, miR-200b-5p, miR-200c-3p, miR-429 and miR-425-5p (**C**).

Next, we compared the mean NGS reads of cell line samples and listed miRNAs that were expressed over two times more in tumor cell lines than in the control cell line. As a result, we obtained a list of 67, 69, 67 and 62 miRNAs expressed at least two times more in HCT116, SW620, SW480 and SW1116, compared to CCD841CoN, respectively. Differentially expressed miRNAs in tumor cell lines were then analyzed using a Venn diagram (Figure 3A), and a list of 45 miRNAs was obtained (Table 1). A similar analysis of miRNAs expressed in tumor EVs was performed. Initial filtering (NGS reads over 30 in all tumor EV samples) revealed 189 miRNAs for further analysis. Then, the fold change of NGS reads of each tumor EV sample vs EVs isolated from the control cell line was calculated, and those miRNAs, whose fold change (FC) was over two, were then analyzed using Venn diagrams (Figure 3B). As a result, we obtained a list of 67, 61, 61 and 62 miRNAs expressed at least two times more in tumor EVs isolated from HCT116, SW620, SW480 and SW1116, respectively, compared to EVs from CCD841CoN. In parallel, we obtained a list of 36 common miRNAs upregulated in all EVs isolated from CRC cell lines. Finally, we analyzed miRNAs for tumor cell lines and their EVs, which allowed us to obtain a list of 22 common miRNAs (Figure 3C). This list included the following miRNAs: miR-196b-5p, miR-429, miR-7-5p, miR-1247-5p, miR-200b-5p, miR-378a-5p, miR-200c-3p, miR-96-5p, miR-200b-3p, miR-9-5p, miR-95-3p, miR-425-5p, miR-200a-5p, miR-203a-3p, miR-17-5p, miR-183-5p, miR-200a-3p, miR-182-5p, miR-1304-3p, miR-577, miR-378a-3p and miR-196a-5p. To investigate the biological role of these miRNAs, KEGG pathway analysis and Gene Ontology (GO) analysis in the category of cellular components was performed. KEGG pathway analysis revealed that up to 15 (miR-7-5p, miR-9-5p, miR-17-5p, miR-96-5p, miR-182-5p, miR-183-5p, miR-196a-5p, miR-196b-5p, miR-200a-3p, miR-200b, miR-200c-3p, miR-203a-3p, miR-378a-3p, miR-425-5p, miR-429 and miR-577) of these miRNAs annotate to proteoglycans in the cancer pathway and the other pathways (PI3K/Akt signaling pathway or cellular senescence), associated with at least 10 of the identified miRNAs (miR-7-5p, miR-17-5p, miR-182-5p, miR-183-5p, miR-196a-5p, miR-196b-5p, miR-200a-3p, miR-378a-3p, miR-429 and miR-577), as shown in Figure 4A. GO analysis of cellular components revealed up to 22 selected miRNAs with significant association in vesicle development and processing (Figure 4B).

From this group, on the basis of high number of reads in the NGS analysis and the common prevalence in at least four top KEGG categories (pathways in cancer, proteoglycan in cancer, the p53 signaling pathway and CRC category), seven miRNAs were chosen for further analysis: miR-9-5p, miR-196b-5p, miR-182-5p, miR-200b-5p, miR-200c-3p, miR-429, miR-425-5p.

Selected seven miRNAs were analyzed using miRNet 2.0 online software to visualize target genes (Figure 4C). We found that five miRNAs from this group (except miR-425-5p and miR-200b-5p) target the BCL2 gene. The next four genes (PTEN, CDKN1B, EP300, SERPINH1) are targeted by at least four miRs; that is: expression of PTEN is regulated by miR-182-5p, miR-200c-3p, miR-425-5p and miR-429, CDKN1B is regulated by miR-182-5p, miR-196-5p, miR-200c-3p and miR-429, and finally SERPINH1 and EP300 are regulated by miR-9-5p, miR-182-5p, miR-200c-3p and miR-429. There is also a group of genes (including, e.g., GSK3b, FOXO1 and VEGF-A, etc.) targeted by at least three from the selected miRs.

**Figure 5:**
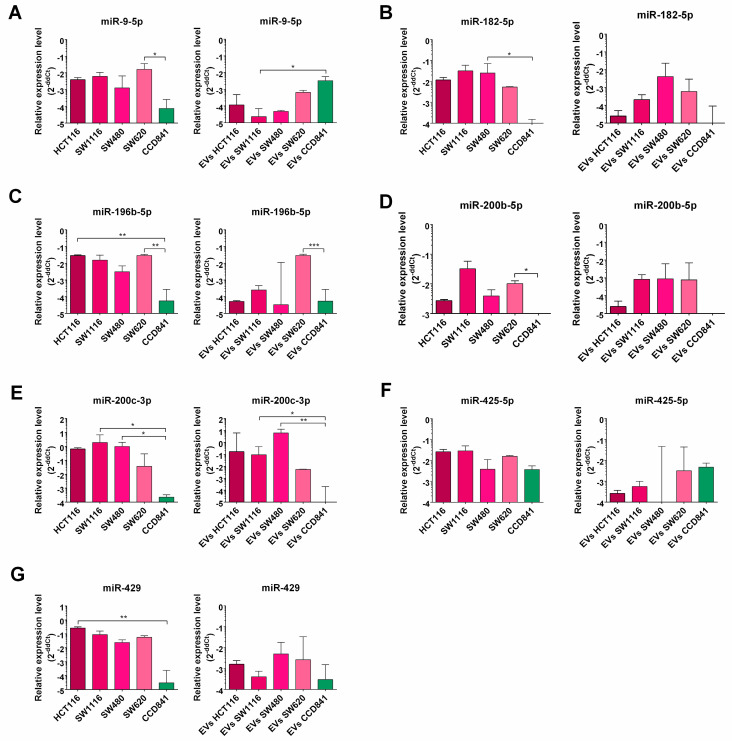
qPCR analysis of selected miRNAs: miR-9-5p (**A**), miR-182-5p (**B**), miR-196b-5p (**C**), miR-200b-5p (**D**), miR-200c-3p (**E**), miR-425-5p (**F**) and miR-429 (**G**), and in all analyzed cell lines and their EVs. miRNA expression was analyzed using RT-qPCR and normalized to U6 expression using the 2^−ΔΔCT^ method. Data were analyzed using the Kruskal–Wallis test with Dunn’s post hoc test, and medians ± IQR are shown. * *p* < 0.05, ** *p* < 0.01, *** *p* < 0.001.

Results of qPCR validation of selected miR expression in cell lines and their EVs are presented in Figure 5. qPCR analysis confirmed that the expressions of miR-196b-5p, miR-429, miR-200b-5p, miR-182-5p and miR-9-5p were significantly elevated in tumor cell lines when compared to the CCD841CoN control cell line. Nonetheless, there were no significant differences in the expression level of miR-425-5p between the analyzed cell lines. The results obtained from the cell lines are similar to EVs miRNA content analysis, except for miR-9-5p, which deviates from this pattern (Figure 5).

In parallel, the most downregulated miRNAs in the CRC cell lines were also analyzed. For this purpose, we selected miRNAs that presented at least 500 reads in the NGS analysis of the miRNA expression profile of the normal CCD841CoN cell line. Then, the fold change of NGS reads count for normal CCD841CoN and median NGS reads count for all CRC cell lines were calculated. The top 30 downregulated miRNAs in the CRC cell lines are presented in Table 1. Among them, two miRNAs were selected—miR-369-3p and miR-143-3p—and their expression was validated in all analyzed cell lines (Figure 2B), confirming the NGS results.

### 3.3. Expression of Selected miRNA in CRC Patients

**Figure 6:**
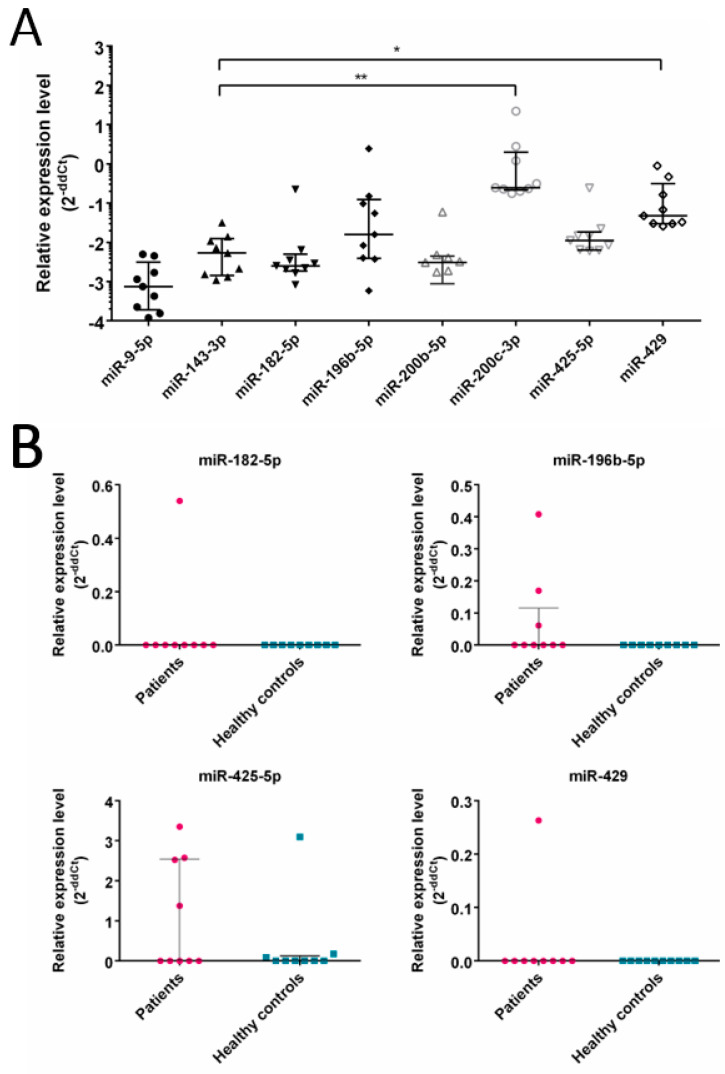
qPCR analysis of selected miRNA expression in CRC patients‘ samples, in comparison to healthy donors. The expression of seven selected miRNAs in tumor samples obtained from CRC patients (**A**). miR-182-5p, miR-196-5p, miR-425-5p and miR-429 expression in EVs isolated from patients’ plasma (**B**). The analysis was performed in CRC patients (n = 9) and healthy donors (n = 9). miRNA expression was analyzed using RT-qPCR and normalized to U6 expression using the 2^−ΔΔCT^ method. Data were analyzed using the Kruskal–Wallis test with Dunn’s post-hoc test (**A**) or Mann–Whitney test (**B**), and medians ± IQR are shown. Asterisks mark significant differences: * *p* < 0.05, ** *p* < 0.01.

Next, the expression of selected miRNAs was tested in tumor tissues of CRC patients to confirm CRC cell line results, and in EVs’ from the plasma of CRC patients to validate possible usage of selected miRNAs as disease biomarkers. We analyzed the expression of seven miRNAs selected above; that is miR-196b-5p, miR-182-5p, miR-200b-5p, miR-200c-3p, miR-9-5p, miR-429 and miR-425, along with miR-143-3p, which was shown to be downregulated in CRC cell lines and their EVs. First, we analyzed the expression of the same set of miRNAs in tumor cell samples obtained from the same nine CRC patients, characterized in Table 2. The results revealed significantly upregulated expression of miR-200c-3p and miR-429 in comparison to normal epithelium cell marker miR-143 (Figure 6A). Then, miRNA expression was determined and compared between EVs isolated from plasma samples collected from nine CRC patients and nine healthy blood donors (Figure 6B). The results showed the expression of only four analyzed miRNAs; that is miR-182-5p, miR-196-5p, miR-425-5p and miR-429, yet the differences between the analyzed groups were not statistically significant. The expression of miR-200b-5p, miR-200c-3p and miR-9-5p were undetectable in any of the patients or healthy controls EVs. All the raw data are presented in Supplementary Table S2.

## 4. Discussion

Changes in miRNA expression have been demonstrated in many types of cancer, including CRC [31]. In the latter, abnormal miRNA expression co-occurs with genetic changes known as the chromosomal or microsatellite instability phenotype and the CpG Island methylator phenotype. One reason for this may be attributed to the fact that half of the miR genes are located in cancer-related regions of the genome or sensitive sites (areas of loss of heterozygosity, cancer-related chromosomal breakpoints or areas of amplification) [32].

The focus of this study was to evaluate miRNA expression in CRC cells and their EVs. We have selected a group of 22 miRNAs that were common to the studied CRC cell lines and were efficiently loaded from tumor cells into their EVs. Our results indicate that some miRNAs were preferentially expressed in normal epithelial cells, with lower or no expression in cancer cells. Finally, the acquired in vitro results concerning miRNA expression were compared to the ones obtained from CRC patients’ samples. The obtained comparison results indicated similarities in miR profile between CRC cell lines and ex vivo tumor tissue. Additionally, some of these miRNAs were also detected in patients’ EVs.

Among the selected 22 miRNAs, seven miRNAs, that is miR-196b-5p, miR-182-5p, miR-200c-3p, miR-429, miRs-425-5p, 200b-5p and miR-9 were considered for further evaluation, on the basis of high number of reads in NGS analysis and common prevalence in at least four top KEGG categories (pathways in cancer, proteoglycan in cancer, the p53 signaling pathway and CRC category).

The miR-196 family consists of three genes that encode miR-196a-1, miR-196a-2 and miR-196b, which produce two mature miRs: miR-196a and miR-196b [33]. Both have been observed to be upregulated in CRC, but miR-196b has been described as the most sensitive and specific marker for detecting this type of tumor [34]. MiR-196b-5p targets genes of the ANXA1 and HOX families in various types of tumors (including CRC), leading to tumor progression [35]. In this study, miR-196b-5p expression in all CRC cell lines and their EVs, as well as in tumor tissue and some plasma-derived EVs, was observed. To our knowledge, the expression of miR-196b in EVs from CRC cell lines has not been previously reported. A significantly higher level of miR-196b was observed in EVs derived from SW620 cells in comparison to EVs from normal colon cells and other tumor EVs. The SW620 cell line was derived from metastasis of the same tumor from which the SW480 cell line was obtained. According to Felizzar et al.’s study, the level of miR-196b-5p varies depending on the stage of the disease, both in tumor tissue and native plasma. In the early stages, the miR-196b-5p expression was lower in tissue and higher in plasma, while the opposite observation was noted in advanced stages, where most of the miR-196b-5p was found in tumor tissues [34]. Our results obtained from the in vitro model (e.g., SW620 vs SW480) confirm Felizzar’s observation; however, the same cannot be said about our in ex vivo evaluation (e.g., a relatively high level of miR-196b-5p in tumor tissues vs low expression in EVs derived from plasma), which may be the consequence of a limited study group number or the analysis of EVs only and not the whole plasma. In our case, miR-196b-5p was detected in EVs derived from only some of the plasma samples of CRC patients (grade 1 or 2). This is in accordance with the studies by Wu et al., where the authors confirmed that, at least in some cases, miR-196b-5p was loaded into EVs. Others have also detected significantly elevated levels of miR-196b-5p in serum exosomes in CRC patients; however, this observation was associated with the occurrence of liver metastases, which we were unable to verify in this study due to the insufficient number of samples from patients with advanced stage of CRC [36]. Our findings, together with the already existing data, support the claims that elevated levels of miR-196b-5p in plasma, whether in its free miRNA or EV-loaded form, may contribute to the detection of cancer.

The miR-183 cluster comprises three miRs: miR-183, miR-182 and miR-96, which are upregulated in CRC patients [37]. It has been observed that the levels of miR-182-5p are elevated in the tumor tissue of CRC patients as compared to non-cancerous tissues [38]. The level of miR-182 is correlated with large tumor size, advanced TNM stage, etc. All tested CRC cell lines expressed miR-182-5p at a high level, resulting in an effective loading into tumor EVs. The normal colon epithelium cell line and their EVs did not express miR-182-5p, which is in line with data obtained from healthy donors’ plasma samples. In our tested tissue samples, the expression of miR-182-5p was relatively low. There may be several possible causes for this observation, e.g., a small group of patients diagnosed in the early stages of cancer, changes in the chromosomal locus (7q31-34) where the miR-182 cluster is located or targeting metadherin, as reported by Jin et al. [39]. In our case, the low expression of miR-182-p in tumor tissue may be the cause of the relatively low expression of miR-182-5p in EVs isolated from the plasma of cancer patients (it was detected in only one out of nine patients). Finally, the expression of free miR-182 in patients’ plasma (but not healthy donors) was reported by Liu et al. [40]. Altogether, monitoring of both forms of CRC-related miR-182-5p (free and loaded into EV) in the blood seems to be important for its correct assessment in early cancer detection.

The miR-200 family comprises five members, namely miR-200a, miR-200b, miR-200c, miR-141 and miR-429. Despite being encoded on two different chromosomes, miR-200b, miR-200c and miR-429 groups share the same seed sequence [41]. These miRs play a crucial role in regulating the epithelial-mesenchymal transition in different types of cancer by repressing the transcription factors ZEB1 and ZEB2 in a bidirectional manner [41]. The high expression of miR-200c and miR-429, as well as the moderate expression of miR-200b in the tested cell lines, mirror their expression in the tumor tissue and are consistent with previously published data [42]. Surprisingly, we did not observe miR-200b-5p and miR-200c-3p to be present in EVs isolated from patients’ plasma. Also, the expression of miR-429 was only detected in one patient, who was classified as G3 with the involvement of 2–3 lymph nodes. Previously, many authors pointed out that a high concentration of miR-200c or miR-200b in serum was associated with the occurrence of lymph node or distant metastases [41,43], which supports our results, because our patients were mainly classified as low-stage, with one G3 exception. Also, Santasusagna et al. presented the limited expression of miR-200 family in vesicles isolated from peripheral veins (far away from the tumor) in comparison to vesicles from the mesenteric vein [44]. This may indicate that the effective loading of the miR-200 family into EVs occurs mainly in the advanced stages of CRC. Moreover, the downregulated expression of miR-429 was reported in the mucosal tissue of patients with inflammatory bowel diseases [45], which supports its CRC relation.

The role of miR-9 depends on the tissue of expression, e.g., oncogenic in cervical or bladder cancer but anti-oncogenic in gastric or ovarian cancer [46]. The function of miR-9 in CRC is ambiguous. Overexpression of miR-9-5p significantly inhibited the proliferation of HCT116 and SW1116 cell lines by targeting the PAK4 kinase [47] and acted as a tumor suppressor by downregulation of BCL-2 and upregulation of BAD [47]. On the other hand, targeting REST resulted in miR-9-induced migration of CRC cells [48]. In this study, miR-9 was detected in all tested cell lines, including normal cells, which is consistent with previous reports [46]. All the tested CRC cell lines harbor a mutation in KRAS [49] that results in the overexpression of miR-9 compared to KRAS-negative cells. In our case, miR-9-5p expression was detected in tumor EVs and EVs from normal cell line, yet its expression was very low in clinical samples, which has been previously reported by others [46] and correlated with poor prognosis [46]. The elevated level of miR-9-5p was observed only in EVs obtained from the normal epithelium cell line, which confirms that miR-9-5p is downregulated in cancer but not in normal cells. This makes miR-9 possibly useful in monitoring CRC patients, but only when their KRAS mutation status is known.

The role of miR-425-5p in promoting CRC by targeting PTEN and modulating the PI3K/AKT/mTOR signaling pathway was proven by Zhao et al. [50]. In addition to Zhao et al.’s report, we have shown that miR-425-5p was loaded into EVs derived from cell lines (including normal epithelium) and detected in CRC patients’ plasma. The high expression of miR-425 in the tumor tissue presented in the current study confirmed previous reports [51]. However, miR-425-5p expression in EVs derived from the serum of CRC patients is unclear. Zhu et al. presented its significant increase, while, on the contrary, Matsumura et al. presented its downregulation synchronized with liver metastasis [51,52]. We observed the presence of miR-425-5p in EVs isolated from four patients and three healthy donors. Its relatively frequent expression may result from the EV source (plasma), which contains significantly more free miR-425-5p than serum (3116 vs. 260 reads) [52,53]. To our knowledge, there are no other reports about the expression of miR-425-5p in EVs derived from CRC patients’ plasma; however, their presence in body fluids in the form of free miR-425 (both isomiRs) has been reported previously [54,55,56]. The detection of miR-425-5p in plasma-derived EVs from CRC patients and healthy donors undermines its diagnostic value and clinical relevance.

In parallel, we have also determined a group of 30 miRs with lower expression in CRC cell lines in comparison to normal cell lines, including miR-143-3p and miR-369-3p, which is in accordance with the data presented by others [57,58]. Moreover, we observed their significant downregulation in tumor EVs. These miRs have been described as a tumor suppressor miRNA responsible for diminishing tumor cell proliferation [57,58]. miR-143 targets directly *KRAS* and *DNMT3A* genes, which results in repression of their translation. The downregulated expression of miR-143 results in aberrant activation of the EGFR pathway [59]. The previously reported presence of KRAS mutation in all tested CRC cell lines may result from the lack of miR-143 in them. However, the presence of miR-143 in tumor tissue may indicate that not all patients were KRAS-positive (KRAS mutations are observed in 40–45% of CRC patients [58] or that the clinical samples were contaminated with KRAS-negative cells.

Our data are consistent with recently published work and provide additional information about the form of miRNA found in the body. We aimed to draw attention to EVs (different types of vesicles) as a common means of transporting miRs that coexist with free RNA in body fluids. Distinguishing between miRNAs in vesicles and RNA attached to other carriers, such as lipo- or ribonucleoprotein, seems to be the nearest/future goal for their reliable and practical use as new cancer markers. Not surprisingly, data from patient plasma-derived EVs do not exactly match data from the cell lines EVs, because patient samples are more complex (composed of miRs from multiple cell populations) compared to miRs obtained from uniform cell cultures. However, carefully designed in vitro experiments can provide valuable information for the selection of appropriate markers for future diagnostic use. Based on our findings, we postulate to consider supplementing the list of previously proposed plasma diagnostic miRs (miR-17-3a, miR-21 miR-29, miR-31, miR-92a, miR-181b, miR-203, miR-221 and let-7g) with [60] miR-182-5p, miR-196b-5p and miR-429, which were found to be expressed in CRC samples but not normal epithelium or in plasma from healthy donors. Our results indicate that the above-mentioned miRs may be considered to be CRC-related.

There are several limitations to our study. Firstly, the efficiency of the total RNA isolation from plasma-derived EVs was low. This may result in the low detection and underestimation of the true level of miRs. Secondly, the study did not present the analysis of free miRNA, which is needed for the assessment of the full picture of miRs circulating in the blood. Thirdly, the analysis of selected miRNAs in EVs isolated from CRC patients was performed in a small group of patients, which may affect our conclusions on the implications of the detection of particular miRNAs. Finally, the selection of miRNAs, based on the results from NGS analysis, largely depended on the analysis strategy and was limited to only the top of the expressed miRNAs. Thus, we cannot exclude that analysis of the expression of other miRNAs in EVs isolated from CRC patients as well as other forms of miRs (e.g., free miRs), would also be clinically relevant.

Concurrently, our study also has several strengths that correlate with the analysis of miRNA profiles of four CRC cell lines, whereas previous reports on this topic focused only on two CRC cell lines, mainly the SW480 and SW620 cell lines [20]. Furthermore, in this work, we have effectively shown similarities in miRNA expression between tumor cell lines and CRC patients’ samples, which strongly legitimizes the concept of the undertaken studies. 

## 5. Conclusions

Our results indicated:

(i) similarities in miRNA expression profile between CRC cell lines (SW480, SW620, SW1116 and HCT116), tumor EVs and patients’ tumor.
(ii) a set of miRs are differentially expressed in CRC cell lines when compared with normal epithelial cell line (CCD841CoN).
(iii) CRC-related miRNAs are efficiently loaded into EVs derived from tested cell lines.
(iv) Several CRC related miRNAs are detected in EVs isolated from CRC patients’ plasma.
(v) Our results extend the previously described profile of CRC-related miRNAs and may become a step toward identifying CRC biomarkers.

## Figures and Tables

**Table 1:** The most upregulated and downregulated miRNAs in the studied CRC cell lines.

Upregulated	Downregulated
miR-7-5p, miR-7-1-3p, miR-9-5p, miR-10a-3p, miR-16-2-3p, miR-17-3p, miR-17-5p, miR-19a-3p, miR-19b-3p, miR-20a-5p, miR-21-3p, miR-25-5p, miR-27a-5p, miR-33b-5p, miR-93-5p, miR-95-3p, miR-96-5p, miR-101-3p, miR-135b-5p, miR-182-5p, miR-183-5p, miR-196a-5p, miR-196b-5p, miR-200a-3p, miR-200a-5p, miR-200b-3p, miR-200b-5p, miR-200c-3p, miR-203a-3p, miR-362-3p, miR-378a-3p, miR-378a-5p, miR-378c, miR-425-5p, miR-429, miR-500a-3p, miR-502-3p, miR-532-5p, miR-577, miR-584-5p, miR-660-5p, miR-1247-5p, miR-1304-3p, miR-12135, miR-12136	miR-127-3p, miR-134-5p, miR-136-3p, miR-143-3p, miR-143-5p, miR-143-5p, miR-154-3p, miR-199a-3p, miR-199a-5p, miR-199b-3p, miR-214-3p, miR-323b-3p, miR-337-5p, miR-369-3p, miR-369-5p, miR-370-3p, miR-382-3p, miR-382-5p, miR-409-3p, miR-409-5p, miR-410-3p, miR-431-5p, miR-432-5p, miR-433-3p, miR-485-3p, miR-490-3p, miR-490-5p, miR-543, miR-665

**Table 2:** The clinical characterization of CRC patients.

Patient	Gender	Age	Localization	Grade	Astler Coller Classification	TNM
1	F	74	sigmoid colon	G2	B2	pT3N0
2	F	36	ascending colon	G1	B2	pT3N0
3	M	67	rectum	G2	C2	pT3N1a
4	M	77	descending colon	G3	C2	PT3N1b
5	F	54	rectum	G1	A	pT1N0
6	F	72	rectum	G1	A	pT1N0
7	M	75	descending colon	G2	C2	pT3N1b
8	M	69	sigmoid colon	G1	B2	pT3N0
9	M	60	rectum	G1	A	pTis

## Data Availability

The data presented in this study are available in the Supplementary Materials (NGS data) or on request from the corresponding author.

## Supplementary Materials

The following supporting information can be downloaded at: https://www.mdpi.com/article/10.3390/cancers16132464/s1, Table S1: NGS results. Table S2: Anonymized qPCR data for CRC patients samples, Figure S1. Expression of EVs markers-Western Blots raw data.
