# Supplementary figures and images for: Identification of miRNAs Present in Cell- and Plasma-Derived Extracellular Vesicles—Possible Biomarkers of Colorectal Cancer
Source: Cancers (Basel). 2024 Jul 5;16(13):2464. doi: 10.3390/cancers16132464 (PMC11240749; doi:10.3390/cancers16132464)

CD9

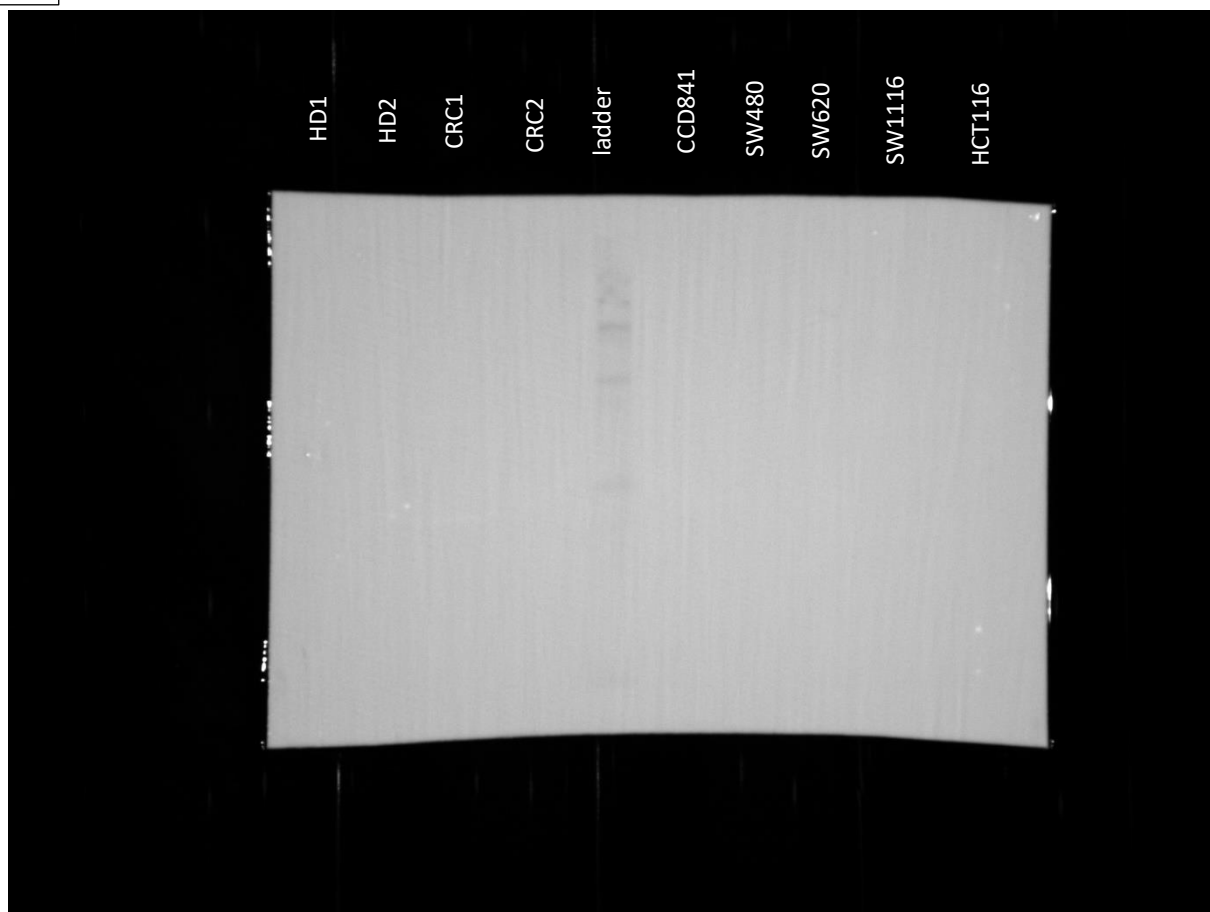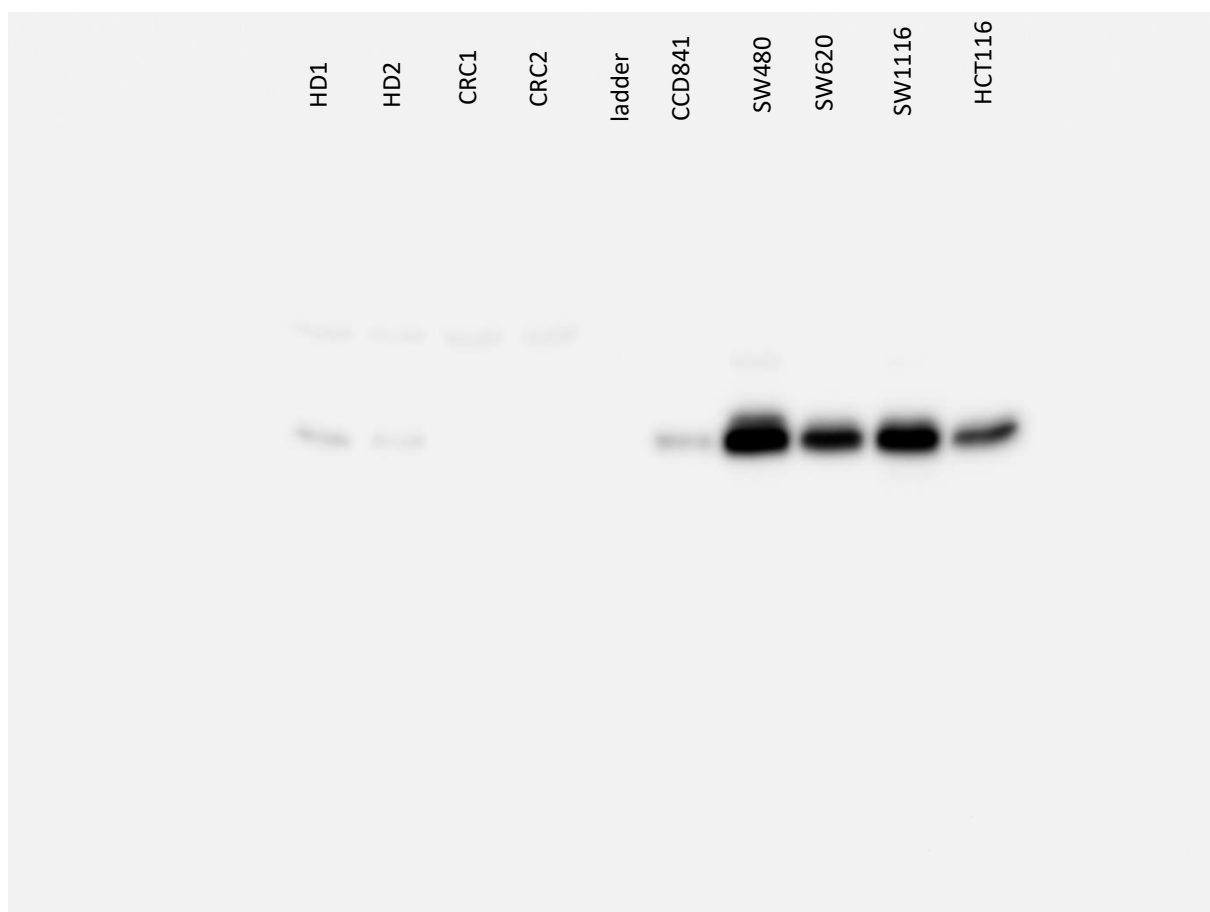

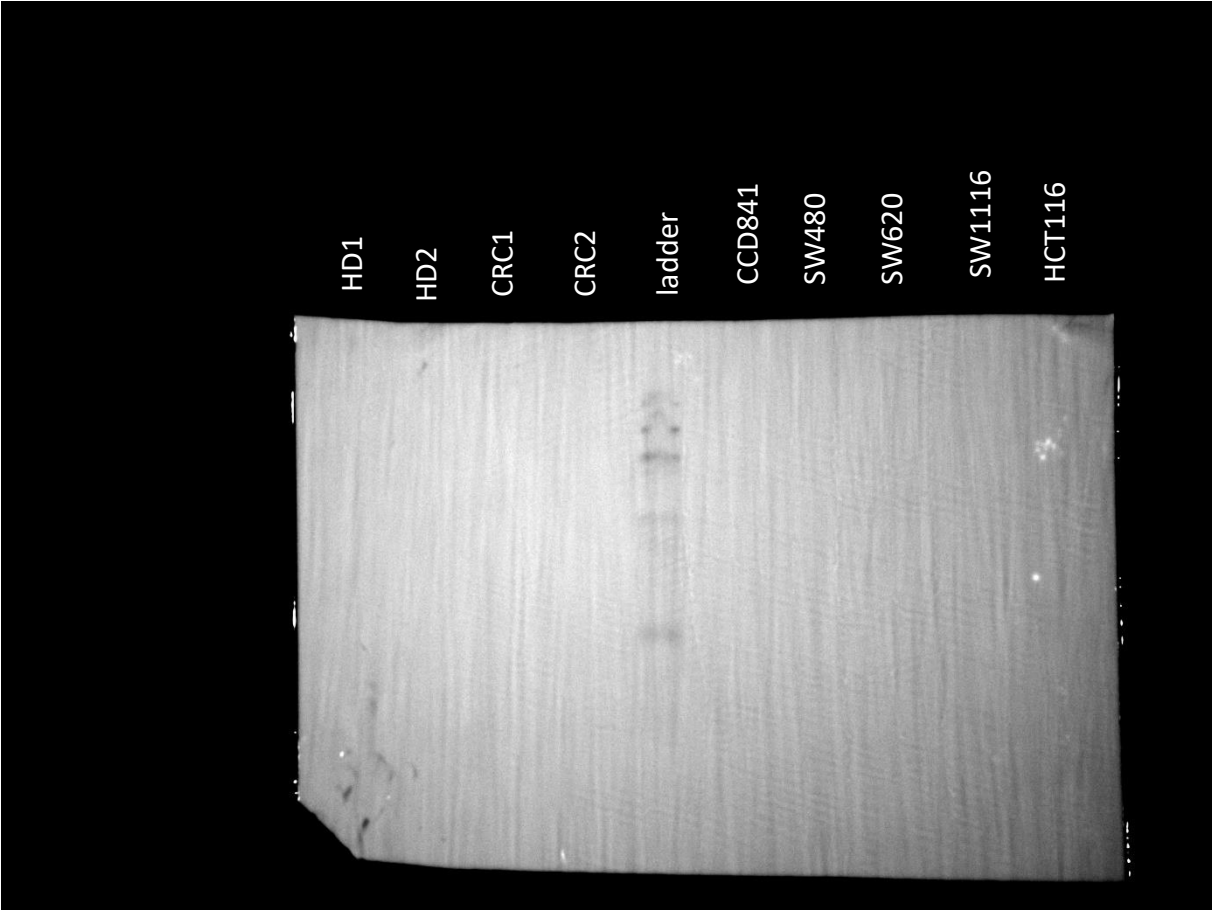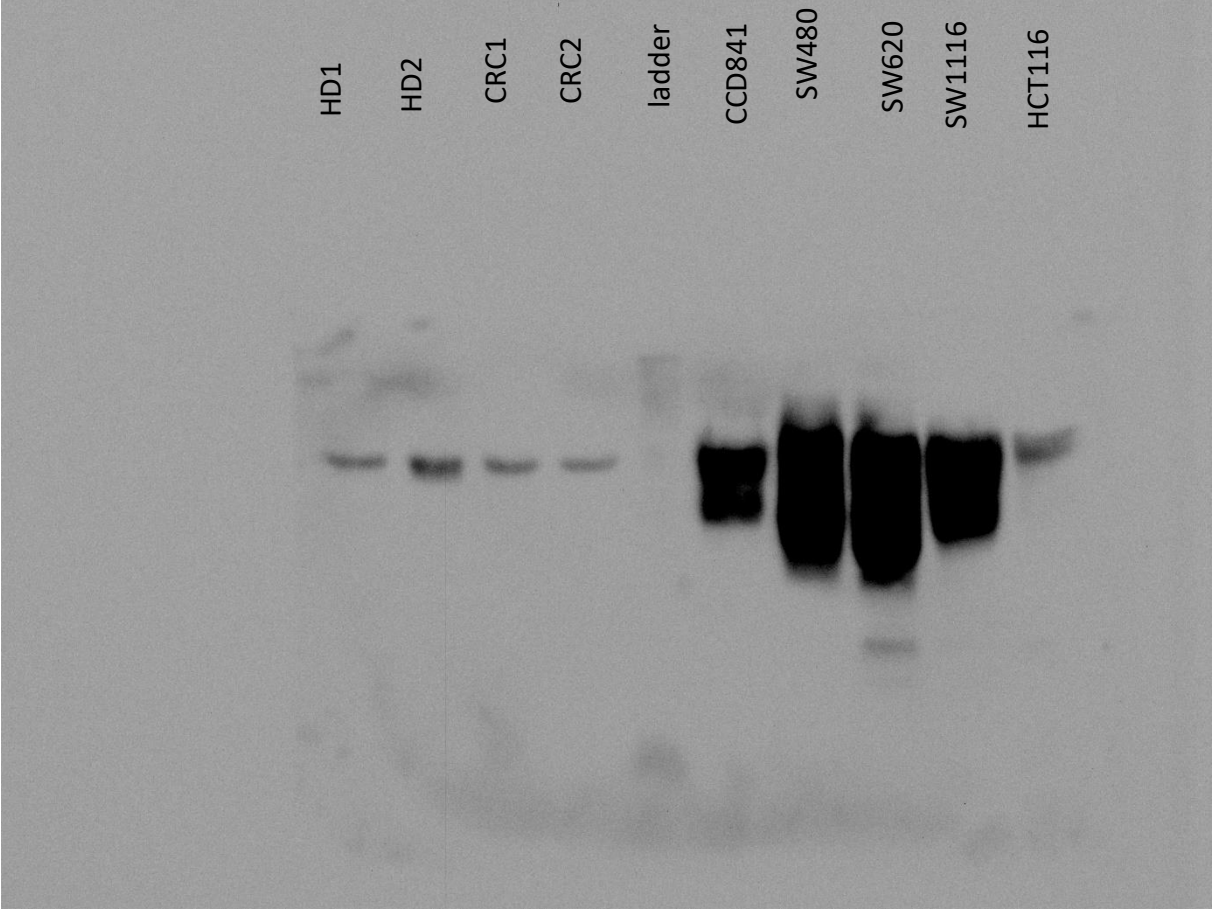

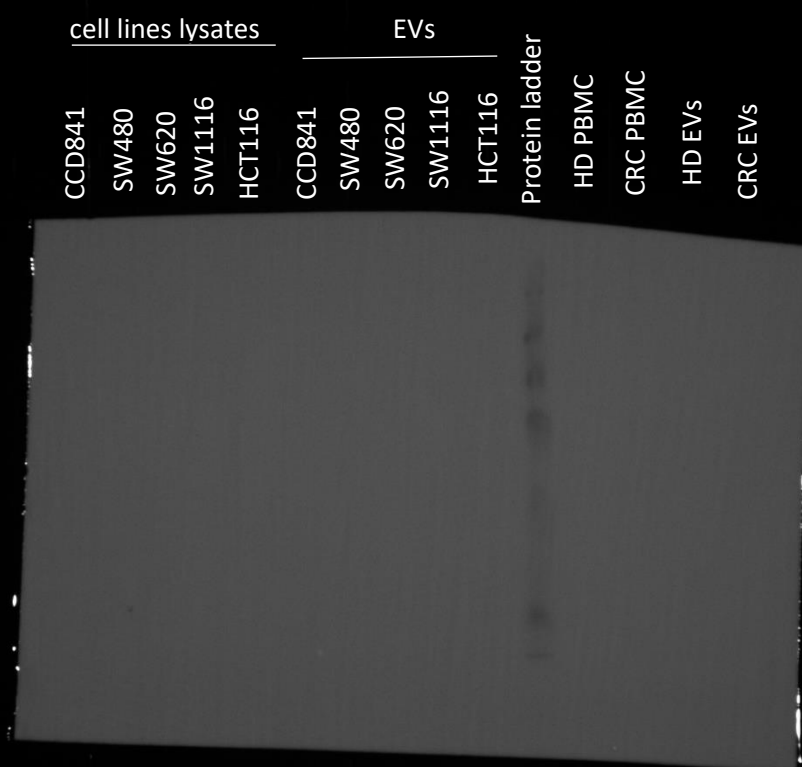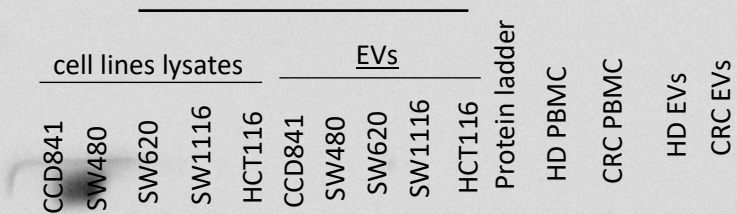

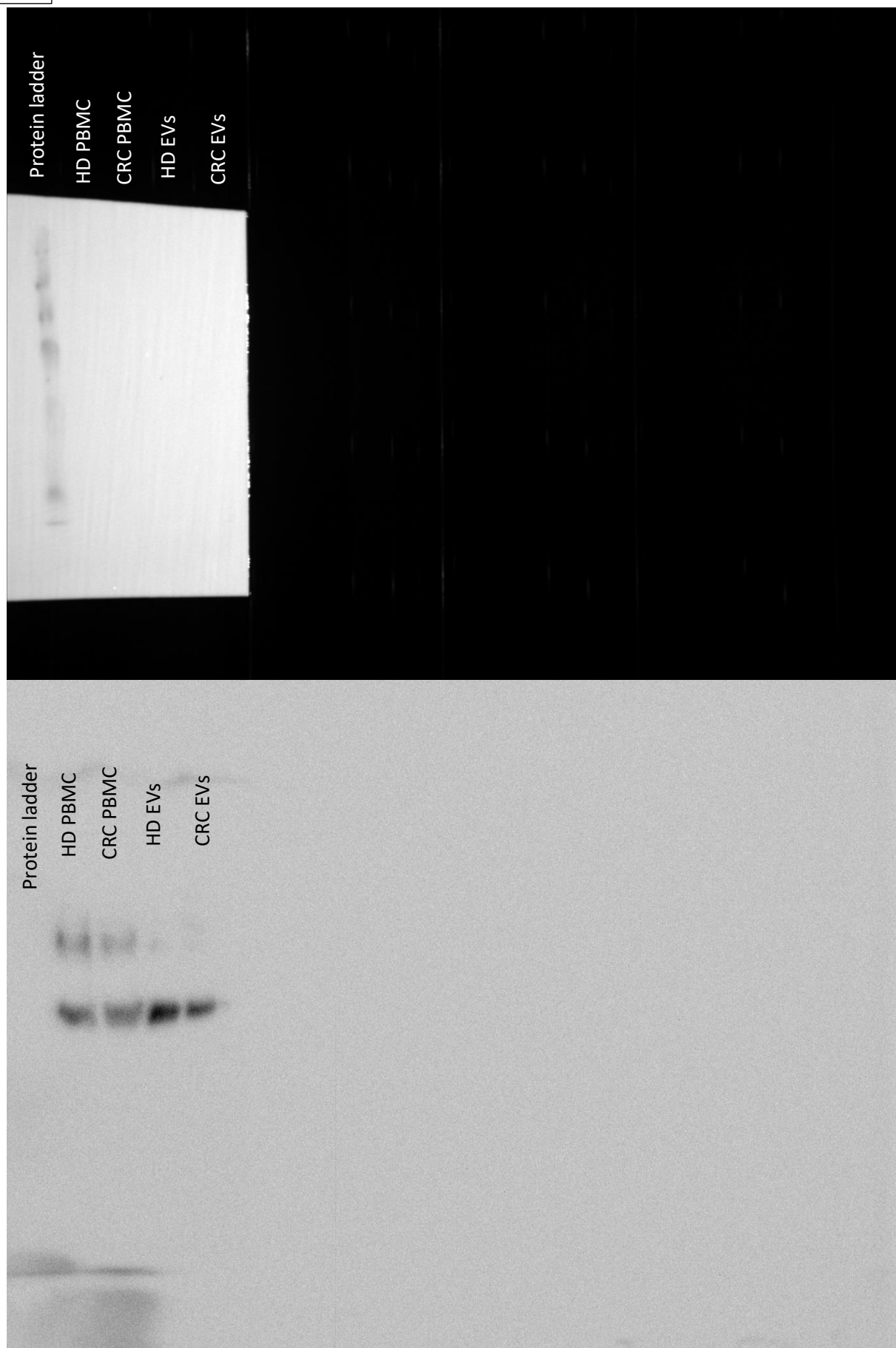

Supplement: Supplementary file 1 [file cancers-16-02464-s001.zip › Figure S1. Expression of EVs markers -Western Blots raw data.pdf]
